# Targeting COVID Vaccine Hesitancy in Rural Communities in Tennessee: Implications for Extending the COVID-19 Pandemic in the South

**DOI:** 10.3390/vaccines9111279

**Published:** 2021-11-04

**Authors:** Donald J. Alcendor

**Affiliations:** 1 Center for AIDS Health Disparities Research, Department of Microbiology, Immunology and Physiology, School of Medicine, Meharry Medical College, 1005 Dr. D.B. Todd Jr. Blvd., Nashville, TN 37208-3599, USA; dalcendor@mmc.edu; 2 Department of Internal Medicine, School of Medicine, Meharry Medical College, 1005 Dr. D.B. Todd Jr. Blvd., Nashville, TN 37208-3599, USA

**Keywords:** COVID-19, Tennessee, rural, urban, vaccine hesitancy, vaccination rate

## Abstract

Approximately 40% of Tennesseans are vaccinated fully, due mainly to higher vaccination levels within urban counties. Significantly lower rates are observed in rural counties. Surveys suggest COVID-19 vaccine hesitancy is entrenched mostly among individuals identifying as white, rural, Republican, and evangelical Christian. Rural counties represent 70 of the total 95 counties in Tennessee, and vaccine hesitancy signifies an immediate public health crisis likely to extend the COVID-19 pandemic. Tennessee is a microcosm of the pandemic’s condition in the Southern U.S. Unvaccinated communities are the greatest contributors of new COVID-19 infections, hospitalizations, and deaths. Rural Tennesseans have a long history of cultural conservatism, poor health literacy, and distrust of government and medical establishments and are more susceptible to misinformation and conspiracy theories. Development of novel strategies to increase vaccine acceptance is essential. Here, I examine the basis of COVID-19 following SARS-CoV-2 infection and summarize the pandemic’s extent in the South, current vaccination rates and efforts across Tennessee, and underlying factors contributing to vaccine hesitancy. Finally, I discuss specific strategies to combat COVID-19 vaccine hesitancy. We must develop novel strategies that go beyond financial incentives, proven ineffective toward vaccinations. Successful strategies for vaccine acceptance of rural Tennesseans could increase acceptance among unvaccinated rural U.S. populations.

## 1. Introduction

Severe acute respiratory syndrome coronavirus 2 (SARS-CoV-2), a newly emerged coronavirus (CoV), has reached pandemic levels since March of 2020 [1,2,3,4]. CoVs are positive-sense, single-stranded, enveloped, RNA viruses [5,6]. These viruses, which belong to the subfamily Coronavirinae, family Coronavirdiae, and order Nidovirales, are classified into four genera: Alphacoronavirus (αCoV), Betacoronavirus (βCoV), Deltacoronavirus (δCoV), and Gammacoronavirus (γCoV) [7,8,9]. As of today, seven human CoVs (HCoVs) have been identified. Of those, two α-CoVs (HCoV-229E and HCoV-NL63) and five β-CoVs (HCoV-OC43, HCoV-HKU1, SARS-CoV, Middle East respiratory syndrome CoV (MERS-CoV), and most recently, β-CoV SARS-CoV-2 (COVID-19)) exist [10,11,12,13,14]. SARS-CoV-2, the virus that causes COVID-19, may produce asymptomatic, as well as severe, acute disease with life-threatening consequences, particularly in individuals with underlying comorbidities [15].

COVID-19 vaccine hesitancy may be defined as a reluctance or unwillingness to be vaccinated or have one’s children vaccinated against the disease, even if proven safe and effective [16,17,18]. Vaccine hesitancy is among the top 10 global health threats identified by the World Health Organization in 2019 [19]. Currently, three COVID-19 vaccines have been approved for emergency use by the U.S. Food and Drug Administration (FDA) and found to be immunogenic, safe, and effective in Phase 3 Efficacy Trials [20,21,22]. In addition, the Pfizer BioN-Tech vaccine has been fully approved for individuals 16 years of age and older by the FDA as of 23 August 2021. However, the prevalence of vaccination hesitancy among communities in Tennessee is high [23]. A survey was conducted in Tennessee with 1000 volunteers focused on beliefs about vaccines and barriers to vaccination, assessed using the validated Vaccine Hesitancy Scale (VHS). The survey focused on historical hesitancy or resistance, perceptions of the vaccine behaviors of others, personal vaccine beliefs, reasons for hesitancy or refusal of external influences on vaccination based on the hesitancy matrix, and potential barriers to vaccination and prior vaccine-related behaviors. Overall, there were positive attitudes toward vaccinations; more than half (54.1%) indicated at least some hesitancy toward vaccination against COVID-19. Regarding hesitancy, 32.1% cited lack of evidence of vaccine effectiveness as the leading reason. Among those that were COVID-19 vaccine hesitant, they identified as moderate or conservative and rural [23]. Several other factors that may contribute to COVID-19 vaccine hesitancy in rural Tennessee communities include fear, conspiracy theories, misinformation/disinformation, as well as the vaccines lacking full approval by the FDA, lack of community health literacy, and distrust in the medical establishment. It has been suggested that the vaccination coverage rate for COVID-19 must reach ~70% to 85% of individuals in the U.S. to achieve herd immunity. Others have suggested that herd immunity is unattainable for COVID-19 in the US and that the virus will eventually become endemic requiring routine scheduled vaccinations [24]. Widespread vaccine hesitancy in rural communities likely will impact these goals and represents an urgent, unmet public health need to curtail or end the COVID-19 pandemic in the U.S. Ongoing COVID-19 vaccine hesitancy exists in rural communities throughout the nation [25]. Several factors contribute to COVID-19 vaccine hesitancy in Tennessee communities, including fear, conspiracy theories, misinformation/disinformation, distrust in government oversight, and associated COVID-19 vaccine side effects, as well as the vaccines lacking full approval by the FDA, lack of community health literacy, and distrust in the medical establishment [23]. We will continue to observe spikes in COVID-19 infections throughout regions of the U.S. among unvaccinated persons due to emergence of COVID-19 genetic variants [26,27,28,29]. Low vaccination rates in Tennessee and other Southern states in the nation will make it difficult to establish herd immunity (~70% to 85% of Americans fully vaccinated) or a level of protection in the general population to limit transmission and severe disease outcomes. Some experts suggest that reaching herd or population immunity is too difficult to achieve due to the continual development of new variants and poor COVID-19 vaccine uptake, particularly in the South. A lack of methods to address widespread COVID-19 vaccine hesitancy in rural Southern communities represents an unmet public health need and is critical for curtailing and, someday, ending the pandemic [30]. To date, only one publication in the PubMed database mentions vaccine hesitancy in the state of Tennessee [23]. Here, I examine the state of vaccine hesitancy among rural populations across Tennessee and provide strategies going forward to improve COVID-19 vaccinations in rural communities.

## 2. COVID-19 Vaccinations in the South

**Figure 1:**
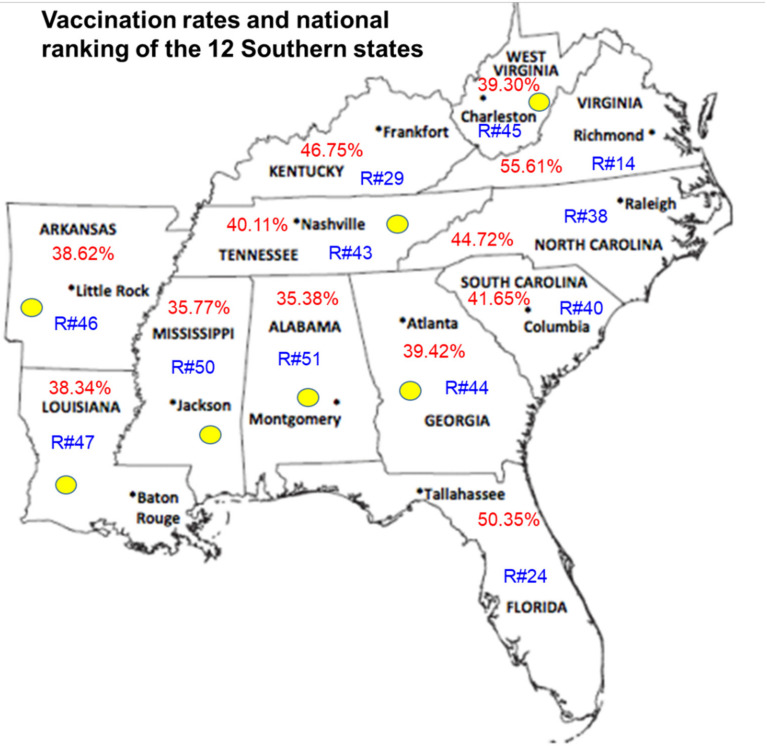
Vaccination rates and national rankings of the 12 Southern states. Vaccination rates within the 12 Southern states are reflected in red text, vaccination rate rankings in the U.S. are reflected in blue text, and states with the lowest vaccination rates in the South are identified with yellow ovals. Vaccination rates were obtained from a statewide https://www.tn.gov/health/cedep/ncov/data.html (accessed on 21 July 2021), vaccine tracker database.

High rates of COVID-19 disease are pervasive in the Southern U.S., which also reflects high rates of unvaccinated communities when compared with those in other regions across the nation. The current pandemic in the US is considered to be a pandemic among the unvaccinated. Southern states have some of the lowest vaccination rates in the U.S [31,32]. (Figure 1). Holding last place, Alabama ranks 51st among states/territories in the U.S. Other Southern states, including Mississippi (50th), Arkansas (46th), Louisiana (47th), Georgia (44th), and Tennessee (43rd) are also are ranked among those states with the lowest fully vaccination rates and the South contains eight of the 10 states with the lowest vaccination rates, according to the CDC (Figure 1) [33]. Poverty, poor health literacy rates, and poor access to healthcare infrastructure has contributed greatly to the low vaccination rates in the South. These low vaccination rates have translated into higher infection rates, more severe disease, increased hospitalizations, and widespread deaths [34]. The Centers for Disease Control and Prevention (CDC) recently reported that as of 26 July 2021, unvaccinated individuals have an 8-fold increased chance of having symptomatic disease if they become infected with the new Delta variant of SARS-CoV-2 and are 25 times more likely to be hospitalized and 25 times more likely to die of COVID-19 disease [35]. The deep-rooted, politically charged cultural conservatism and misinformation campaigns about COVID-19, the pandemic, and emerging vaccines are widespread in the South and must be addressed at a scale that includes trusted messengers from these communities. However, trusted members from faith-based communities in the South, including prominent pastors, often have disregarded the COVID-19 pandemic as a hoax and the vaccines as a scam when speaking to their parishioners [36]. Access to testing and vaccinations is no longer a significant barrier for Southern communities. Testing and vaccination venues now include grocery stores, barbershops, salons, drugstores, and churches, along with mobile vaccine units that meet people where they are to deliver COVID-19 testing and vaccination services [37].

## 3. COVID-19 Vaccinations in Tennessee

**Figure 2:**
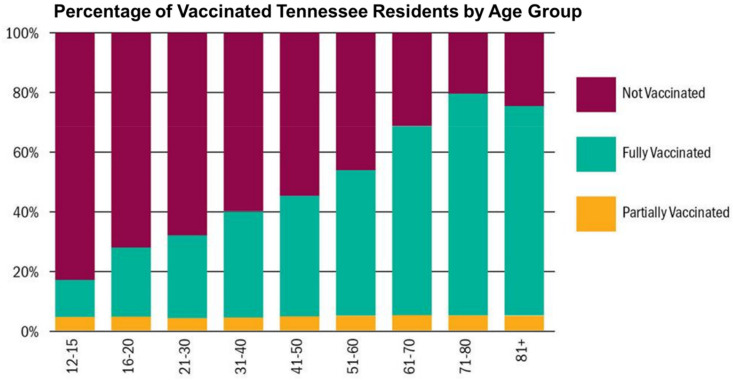
Percentage of Tennesseans unvaccinated, fully vaccinated, and partially vaccinated by age group as of 11 July 2021. The data shows the percentage of individuals vaccinated and unvaccinated by age. The percentage of residents who remain unvaccinated appears as red bars. The percentage of residents who are vaccinated fully appear as green bars, and those partially vaccinated appear as yellow bars (Source: *Tennessee Department of Health* https://www.tn.gov/health/cedep/ncov/data.html; accessed on 11 July 2021).

**Figure 3:**
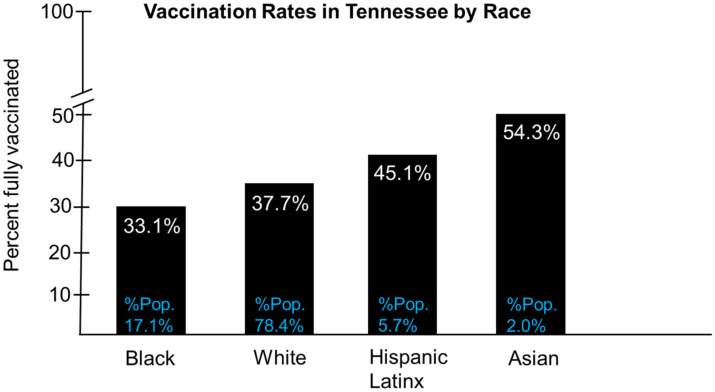
Vaccination rates in Tennessee by race. Vaccination rates in Blacks, Non-Hispanic whites, Hispanic/Latinx, and Asians appear as the percentage of the population they represent (Source https://www.tn.gov/health/cedep/ncov/data.html (accessed on 11 July 2021).

As of 16 August 2021, a total of 168,362,058 Americans, or 50.7% of the country’s population, have been vaccinated fully, according to the CDC [38]. Tennessee ranks 43rd out of 50 states (51 including the District of Columbia), having a total of 2,739,215 Tennesseans fully vaccinated, which represents 40.11% of the state’s total population [38]. Financial incentives to improve vaccine uptake in the Southern states, such as Tennessee, have resulted in no lasting change in COVID-19 vaccine acceptance. COVID-19 vaccines have been highly politicized in Tennessee and proposed vaccine mandates could lead to increased resistance and possible violent behaviors. The number of unvaccinated Tennesseans remains high. The establishment of the Delta variant as the dominant SARS-CoV-2 strain in the U.S. is currently responsible for a 4th surge in new infections, hospitalizations, and deaths, particularly in rural communities. Therefore, it is essential to vaccinate more Tennesseans. Vaccination among the elderly in Tennessee, ages 61 to 81+, has resulted in reduced COVID-19-related hospitalizations and mortality [39]. However, the percentages of unvaccinated Tennesseans aged 12 to 15, 16 to 20, 21 to 30, and 31 to 40 are approximately 88%, 70%, 65%, and 60%, respectively (Figure 2) [39]. The high level of unvaccinated individuals has led to increased infection rates and hospitalizations among these age groups [39]. The percentages of unvaccinated individuals among age groups 41 to 50 and 51 to 60 years are approximately 55% and 45%, respectively (Figure 2) [39]. Approximately 5% of all Tennesseans are vaccinated partially for COVID-19 (Figure 2) [39]. Due to the recent rise of the Delta variant, particularly in Tennessee, patient age demographics for hospitalizations due to COVID-19 have changed to reflect an increase among younger individuals, including teenagers and children [40]. In this study, current COVID-19 vaccination rates are examined by race and population (Figure 3). Specifically, Blacks and non-Hispanic whites have the lowest vaccination rates in Tennessee at 33.1% and 37.7%, respectively, and represent 17.1% and 78.4% of the state’s population, respectively (Figure 3) [41]. Hispanic/Latinx communities have a combined vaccination rate of 45.1% and represent 5.7% of the population (Figure 3). Finally, Asians have the highest vaccination rate at 54.3% and represent only 2.0% of the population in Tennessee (Figure 3) [41].

### 3.1. Urban Communities

**Figure 4:**
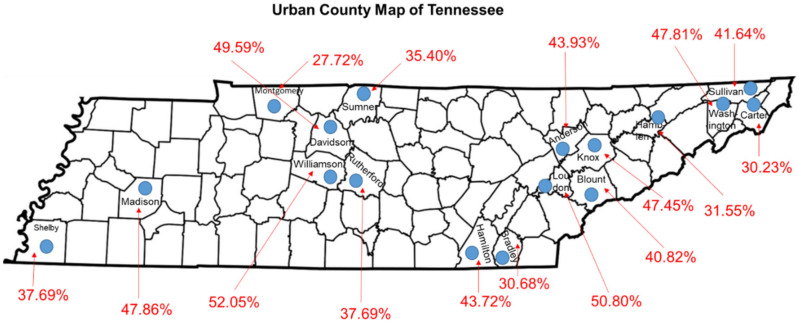
Vaccination rates in the 17 urban Tennessee counties. Urban counties are labelled in black and designated as blue ovals. Vaccinations are given as percentages in red text. Vaccination rates were obtained from the Tennessee Department of Health. https://www.tn.gov/health/cedep/ncov/data.html (accessed on 11 July 2021).

In urban Tennessee communities, COVID-19 vaccinations have fallen short of expectations, but the rates of vaccinations in these areas have outperformed rates observed in rural communities [42]. Urban communities in Tennessee have greater access to COVID-19 testing and vaccinations. These counties also are large and more industrialized, with greater access to the healthcare infrastructure, have increased opportunities to acquire health literacy, have progressive ideas regarding new technologies, and tend to be more forward thinking as it relates to health and health policies. Urban communities are better equipped to care for patients with COVID-19, along with having more staff to accommodate higher patient loads [43]. The 17 urban counties in Tennessee have significantly higher population densities, more advanced learning centers, and more advanced medical infrastructure compared with the 78 rural counties (Figure 4) [44]. These densely populated urban counties also have significantly higher vaccination rates than the rural counties (Figure 4) [44].

### 3.2. Rural Communities

Rural communities in Tennessee tend to be more undeveloped with large areas of farmland, and community members often reside outside of industrial centers and must travel long distances to gain access to COVID-19 testing and vaccinations. In general, these communities have strong, negative feelings toward the COVID-19 vaccines, and residents are prepared to become unemployed when faced with masking and COVID-19 vaccine mandates implemented at work. Residents within these communities often are older, have lower literacy rates when compared with those in urban communities in Tennessee, and have fewer opportunities to gain access to health literacy regarding COVID-19 disease and the resulting vaccines. Not having trust in the government appears to be the overwhelming reason why rural community members refuse to get the vaccine [45]. Rural residents who are distrustful of the vaccines are less likely to follow CDC guidelines, such as masking and social distancing during large public gatherings. Even more, distrust of the COVID-19 vaccines by adults from rural communities in Tennessee will likely lead to poor vaccine uptake by family members and their children leading to widespread viral transmission in schools and the general population at large. However, children 11 and younger are not yet eligible for the COVID-19 vaccine. Taken together, this escalating series of events could result in more government shutdowns in Tennessee and other states with low vaccination rates in rural communities.

**Figure 5:**
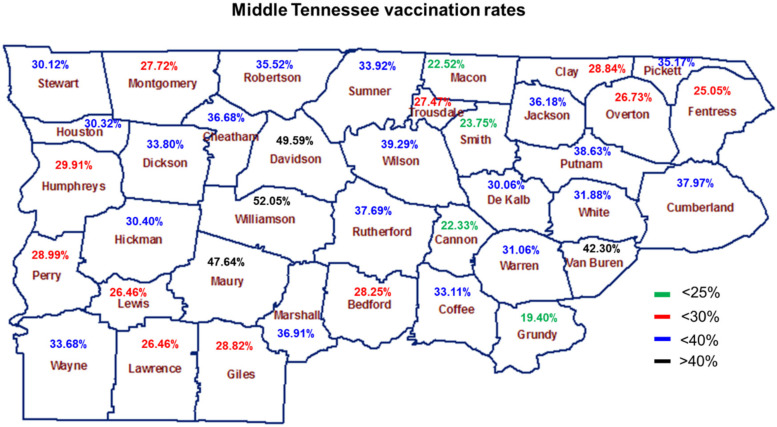
Vaccination rates for the 38 counties in Middle Tennessee as of 18 July 2021. Counties in Middle Tennessee are labeled in brown text. Vaccination rates < 25% are shown in green text, vaccination rates < 30% are shown in red text, vaccination rates < 40% are shown in blue text, and vaccination rates > 40% are shown in black text. Vaccination rates were obtained from the Tennessee Department of Health https://www.tn.gov/health/cedep/ncov/data.html (accessed on 11 July 2021).

Vaccination rates in Middle Tennessee are shown in Figure 5. The percentage of unvaccinated rural Tennesseans is significantly higher than that of unvaccinated Tennesseans residing in urban communities (Figure 5). Although urban counties near Nashville (Davidson County) have significantly higher vaccination rates (denoted in black), rural counties, such as Macon, Montgomery, Hickman, Giles, Cannon, Bedford, and Smith, have significantly lower vaccination rates (denoted in blue, red, and green) (Figure 5). Vaccination percentages represent populations that are vaccinated fully.

## 4. Factors Contributing to COVID-19 Vaccine Hesitancy in Rural Tennessee Counties

According to the U.S. Census Bureau, 78.4% of the state of Tennessee’s population is white, 17.1% is African American, 5.7% is Hispanic/Latinx, 2.0% is Asian, 0.5% is American Indian or Alaska Native, and 0.1% is Native Hawaiian or Other Pacific Islander. Based on the 2010 Census, 93% of Tennessee is rural based; 78 of 95 counties had at least 50% of their residents living in the 38,330 square miles of rural Tennessee. These rural communities are culturally conservative and are prone to accept misinformation, adapt to fear and conspiracy theories, lack vaccine awareness and health literacy, have antivaxxer beliefs, and have politicized COVID-19 vaccines [46]. Many also have safety concerns, such as side effects associated with the vaccines, and many have decided to wait until the vaccines have been approved fully by the FDA. Rural communities also face extended travel time to an acute care facility, hazardous terrain, and the lack of reliable or public transportation. These challenges may cause patients to postpone or go without accessing health services. In addition, the health infrastructure in rural communities is not adequate for large-scale care of COVID-19 patients. The CDC reports more than 170 hospitals in rural America have closed since 2005 and another 700 are at risk of closure [47]. Rural communities have a longstanding shortage of primary care providers. Rural health systems and providers will have a difficult path forward facing expanding budget shortfalls, staff shortages, and a lack of infrastructure to support a growing COVID-19 patient population. This will require comprehensive federal programs to support the needs of rural health systems and providers impacted by the COVID-19 pandemic. In addition, CDC reports suggest that poor overall health of the population in rural America, inadequate healthcare infrastructure, and dependence on the existence of agricultural and food processing industries in rural areas place these populations at higher risk of COVID-19 acquisition and the most severe symptoms of the disease, resulting in poor clinical outcomes. Moreover, rural Tennesseans are less likely to view the COVID-19 pandemic as a serious threat, either to the U.S. or their families. Currently, nearly all COVID patients who are hospitalized or die of COVID-19 disease are unvaccinated. Tennessee ranks 43rd out of 50 states, with only 40.11% of Tennesseans completing vaccinations. Lower vaccination rates exist in rural communities throughout the state when compared with urban communities, suggesting an urgent, unmet public health need to increase COVID-19 vaccine uptake among these populations.

## 5. COVID-19 Vaccine Hesitancy in Urban Communities

In urban areas, the profile of vaccine-hesitant individuals tends to differ. Although only 17 counties in Tennessee are urban, 5.3 million of the 6.8 million residents in the state live in these urban areas based on 2010 census data. Approximately 1.5 million residents live in rural Tennessee counties and have a significantly lower vaccination rate when compared with urban counties. The reasons for vaccine hesitancy in urban communities tend to overlap with reasons observed in rural communities but to a different degree. Urban community members tend to be more educated about the COVID-19 pandemic and resulting vaccines and have greater access to health services, increased opportunities to access health literacy, and greater access to broadband internet services to facilitate their understanding of COVID-19 and the existing vaccines. However, racial and ethnic minorities residing in urban Tennessee counties also are distrustful of government and medical establishments for historic reasons. These underserved communities continually are subjected to institutionalized racism and social and economic inequities. Residents also face poor social determinants of health, which are long-standing and have contributed greatly to widespread vaccine hesitancy among rural and urban African American and Hispanic/Latinx populations across the state.

## 6. Specific Strategies to Combat COVID Vaccine Hesitancy in Rural Tennessee Communities

The misinformation surrounding vaccine efficacy and safety must be addressed in rural communities using CDC/FDA guidance delivered at a culturally competent level of understanding by trusted members within these communities. Interactions must occur via places in which rural residents come together as groups, such as barbershops, salons, grocery stores, feed and supply stores, and faith-based community settings. We must provide rural community members with opportunities to achieve a level of health literacy to help them understand the basics of COVID-19 disease and the purpose of vaccines in preventing infection. To bring our COVID-19 vaccine message to scale in rural areas and reach a larger audience, we need extensive collaboration with members of these communities and, even more, celebrities who are supportive of the COVID-19 vaccines. Information could be disseminated widely at social events, including public gatherings and those occurring within academic settings, via Town Halls, via social media platforms, including Facebook Live and Instagram, and via radio broadcasting and local news outlets. Country and western music is enjoyed by rural residents throughout Tennessee, and many Tennesseans view country music celebrities as personally influential. These celebrities may be recruited to develop specific messaging and to use their respective platforms to support COVID-19 vaccine acceptance. In addition, sports personalities interested in promoting vaccine acceptance may participate in radio and television advertisements and attend specialty events, such as the Harvest Festival and Tennessee State Fair.

Community representation in the effort to improve COVID-19 vaccination uptake in rural populations across Tennessee is essential. In addition, it is believed that trusted messengers from these rural communities will be most effective in providing information on COVID-19 vaccine efficacy and safety to their own communities at large. Collaborative efforts are required to recruit rural residents across the age spectrum to support COVID vaccine acceptance in counties with low vaccination rates. Both young and elderly family members have great influence on decision making within households. To aid vaccine acceptance efforts, individuals within communities would be designated COVID-19 vaccine ambassadors. To become an ambassador, an individual must be vaccinated fully at the time of recruitment. The role of ambassadors would be to provide their personal perspectives on getting the vaccine, including how being vaccinated is better than being unvaccinated. Culturally appropriate COVID-19 awareness and prevention information also must be provided to underrepresented ethnic and racial minorities residing in rural Tennessee, such as community members who have English as a second language. Comprehensive studies designed to target rural Tennessee communities experiencing vaccine hesitancy will lead to increased COVID-19 vaccination rates and a model for improving overall vaccine acceptance rates in rural communities throughout the US.

To ensure equitable COVID-19 vaccine access for rural community members, mobile vaccination units may be employed to target these areas with low vaccination rates. Mobile vaccine units are able to deliver COVID-19 vaccines to rural residents who are mobility challenged or homebound and who are at higher risk for the more severe symptoms of COVID-19.

## 7. Outlook for Rural Communities in Tennessee during the Course of the Pandemic, Changes, and Projected Consequences

The COVID pandemic is surging in unvaccinated communities throughout the U.S. Unvaccinated communities represent a reservoir of infection that will feed this pandemic over time, resulting in increased infections, hospitalizations, and deaths. The expanded pandemic will contribute to continual evolution of genetic variants that could be more resistant to the protection provided by existing vaccines. Increased COVID-19 vaccination acceptance rates of rural communities could lead to increased vaccine acceptance rates for commonly transmitted diseases, resulting in reduced morbidity and mortality in the general population, and thereby leading to increased productivity due to fewer sick days and reduced healthcare costs. Although COVID-19 vaccine access in the US has greatly improved, vaccine access for the underserved such as the homeless and immigrant populations is still problematic. Travel to pharmacies and vaccine centers along with language barriers can greatly impact these communities for vaccine acceptance and uptake. The success of COVID-19 vaccines will depend on high vaccine acceptability and organized strategies to combat lack of education regarding vaccines and vaccine misinformation. Finally, such strategies must be delivered in a culturally competent manner to rural Tennessee communities.

## 8. Conclusions

The pandemic has impacted the global economy and threatened the health care systems in the US. Methods to address widespread COVID-19 vaccine hesitancy in rural communities across the state of Tennessee have been elusive, representing an urgent, unmet public health need that is essential for curtailing or ending the pandemic. Comprehensive studies designed to target rural Tennessee communities experiencing vaccine hesitancy are needed and could provide a model for improving overall COVID-19 vaccine acceptance rates in rural communities throughout the South. Increased uptake in rural communities for other important vaccines, such as influenza, pneumococcal, varicella, and human papillomavirus (HPV), could follow. Overall increased vaccination acceptance rates for commonly transmitted diseases could result in reduced morbidity and mortality within the general population. In addition, it is understood that we cannot demonize members of rural and urban communities for deciding not to accept the COVID-19 vaccine. Surveys conducted on vaccine hesitancy in Tennessee find that these individuals mostly identify as white, rural, Republican, and evangelical Christian [48]. Cultural conservatism in the South could lead to division among the vaccinated and the unvaccinated to accept or refuse a vaccine that remains the best option for protection against severe disease and death due COVID-19. These factors may greatly contribute to the low vaccination rates throughout the South. COVID-19 vaccine hesitancy exists throughout the entire U.S., and therefore, we must address this problem at scale to achieve the greatest impact. The future is uncertain because of the continual evolution of the virus along with breakthrough infections to existing vaccines and long-lasting poor vaccination rates especially in rural communities throughout the US.

## Data Availability

Additional data for this study may be found via the Tennessee Department of Health Epidemiology and Surveillance Data vaccine tracker database (Source https://www.tn.gov/health/cedep/ncov/data.html; accessed on 16 August 2021).
